# ERICH3 in Primary Cilia Regulates Cilium Formation and the Localisations of Ciliary Transport and Sonic Hedgehog Signaling Proteins

**DOI:** 10.1038/s41598-019-52830-1

**Published:** 2019-11-11

**Authors:** Mona Alsolami, Stefanie Kuhns, Manal Alsulami, Oliver E. Blacque

**Affiliations:** School of Biomolecular and Biomedical Science, University College Dublin, Belfield, Dublin 4 Ireland

**Keywords:** Cilia, Protein translocation

## Abstract

Intraflagellar transport (IFT) is essential for the formation and function of the microtubule-based primary cilium, which acts as a sensory and signalling device at the cell surface. Consisting of IFT-A/B and BBSome cargo adaptors that associate with molecular motors, IFT transports protein into (anterograde IFT) and out of (retrograde IFT) the cilium. In this study, we identify the mostly uncharacterised ERICH3 protein as a component of the mammalian primary cilium. Loss of ERICH3 causes abnormally short cilia and results in the accumulation of IFT-A/B proteins at the ciliary tip, together with reduced ciliary levels of retrograde transport regulators, ARL13B, INPP5E and BBS5. We also show that ERICH3 ciliary localisations require ARL13B and BBSome components. Finally, ERICH3 loss causes positive (Smoothened) and negative (GPR161) regulators of sonic hedgehog signaling (Shh) to accumulate at abnormally high levels in the cilia of pathway-stimulated cells. Together, these findings identify ERICH3 as a novel component of the primary cilium that regulates cilium length and the ciliary levels of Shh signaling molecules. We propose that ERICH3 functions within retrograde IFT-associated pathways to remove signaling proteins from cilia.

## Introduction

Primary cilia are microtubule-based organelles extending from the surfaces of most mammalian cell types. The canonical ciliary axoneme is comprised of a cylinder of 9 doublet microtubules extending from a mother centriole-derived basal body, enveloped by a contiguous and specialised extension of the plasma membrane^1^. Primary cilia act as critical sensory devices that receive and transduce a wide range of physical and chemical stimuli such as light, temperature and mechanical cues. Cilia also coordinate several important extrinsic signalling pathways (eg. Sonic hedgehog (Shh), TGF-beta, Wnt) that regulate many important aspects of cell genesis, differentiation and behaviour, both in the embryo and adult^2^. Defects in ciliary structure and function lead to pleiotropic, overlapping and broad spectrum diseases termed the ‘ciliopathies’ that affect the development and function of many tissues and organs^3^. Examples include Bardet-Biedl syndrome (BBS), characterised amongst others by blindness, obesity, kidney dysfunction, bone abnormalities and intellectual disability, as well as Joubert Syndrome (JBTS), which presents with many of the aforementioned abnormalities along with cardinal features of mid-hindbrain malformation^4,5^.

The formation and maintenance of primary cilia is critically dependent on the cycling intraflagellar transport (IFT) system, which operates bidirectionally along the ciliary microtubules to deliver proteins to, and remove proteins from, the organelle. Driven by kinesin-2 anterograde (towards ciliary tip) and IFT-dynein retrograde (towards ciliary base) motors, IFT trains possess multisubunit IFT-A and IFT-B particle complexes that serve as import and export adaptors for ciliary protein cargo^6,7^. The IFT-A complex (6 proteins) is biochemically organised into core (IFT122/140/144) and non-core (IFT43/121/139) submodules, and regulates the ciliary tip formation of cargo-laden retrograde IFT trains, as well as the ciliary import of at least some membrane proteins (eg. GPCRs)^8–22^. The larger IFT-B complex (16 proteins) consists of core B1 (IFT22/25/2746/52/56/70/81/88) and peripheral B2 (IFT20/38/54/57/80/172) subcomplexes, with roles in anterograde IFT train assembly and the ciliary import of proteins such as tubulin subunits and motility-associated proteins (eg. inner/outer dynein arms)^22–31^. A third IFT cargo adaptor is the eight membered BBSome (BBS1/2/4/5/7/8/9 and BBIP10), which is associated with membrane protein removal from cilia via retrograde IFT^32–40^. Also, at least in sensory neuronal cilia, the BBSome coordinates IFT-A/B complex trafficking^41–43^. Various regulators for IFT are known including the ciliary membrane-associated G protein ARL13B, which biochemically interacts with IFT-B and stabilises anterograde IFT in *C. elegans* and retrograde IFT in mammalian cells, and is required for the targeting and distribution of ciliary membrane proteins such as the PI(4,5)P_2_ phosphatase INPP5E^44–50^.

Primary cilia are critically important for Shh signaling, where all of the key pathway components associate with the organelle, including the Patched1 (PTCH1) receptor, positive (Smoothened; SMO) and negative (GPR161; SUFU) pathway regulators, and downstream Gli transcription factors that get processed to activator (GliA) or repressor (GliR) forms^51–56^. During pathway activation, ligand binding causes PTCH1 to exit the cilium and SMO to accumulate in the cilium, resulting in GliA formation^52,53^. GliA build-up is also promoted by ligand-induced ciliary removal of the negative pathway regulator, GPR161^57,58^. The dynamic movement of Shh signaling components into and out of cilia, and therefore pathway output, is heavily reliant on IFT^11,59–65^. For example, TULP3-mediated targeting of GPR161 to cilia occurs via direct interaction with the IFT-A core complex^10,13^. In contrast, the ciliary removal of GPR161, as well as SMO and PTCH1, depends on the IFT-A non-core complex, IFT25/27 (distinct submodule of IFT-B1) and the BBSome^13,35–38,40,63,66^. ARL13B and one of its proposed effectors, INPP5E, also promote the ciliary removal of GPR161, and regulate ciliary targeting of SMO^46,50,67–70^.

In this study we identify the mostly uncharacterised protein ERICH3 (Glutamate rich protein 3) as a new component of the primary cilium. By employing the human hTERT-RPE1 cell line and siRNA-mediated depletion, we show that ERICH3 positively controls cilium formation and length, and limits the ciliary levels of SMO and GPR161 in pathway-stimulated cells. ERICH3 also promotes BBSome, ARL13B and INPP5E enrichment in cilia and prevents the ciliary tip accumulation of IFT particle proteins. Together, our findings implicate ERICH3 as a novel component of retrograde IFT-associated pathways that remove Shh signaling regulators from cilia.

## Results

### ERICH3 localises to primary cilia

**Figure 1:**
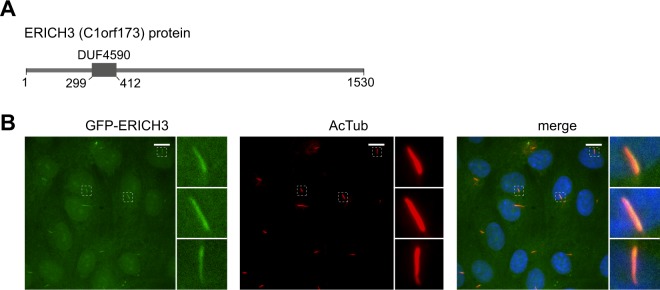
ERICH3 localises to the primary cilium. (**A)** Schematic of the ERICH3 protein showing the location of the DUF4590 domain. Numbers refer to amino acid positions. **(B)** Representative images of stably expressed GFP-ERICH3 in hTERT-RPE1 cells after 48 h serum starvation, stained for cilia (acetylated tubulin; red) and the nucleus (DAPI; blue). Scale bars; 10 µm. Small panels are higher magnification images of the boxed regions.

Recently, we employed a co-expression approach to identify candidate ciliary genes in humans and mice^71^. One of the top hits within these datasets is C1orf173, also known as ERICH3 (Glutamate rich protein 3), which encodes a large protein (1530 amino acids) containing a 113 amino acid domain of unknown function (DUF4590) (Fig. 1A). Although ERICH3 is linked to osteoporosis and colorectal cancer^72^, as well as the regulation of plasma serotonin concentration^73^, the cellular roles of this protein remain almost completely unknown. Interestingly, ERICH3 was identified as an abundant ciliary protein in a recent proteomics study of isolated human airway motile cilia^74^.

**Figure 2:**
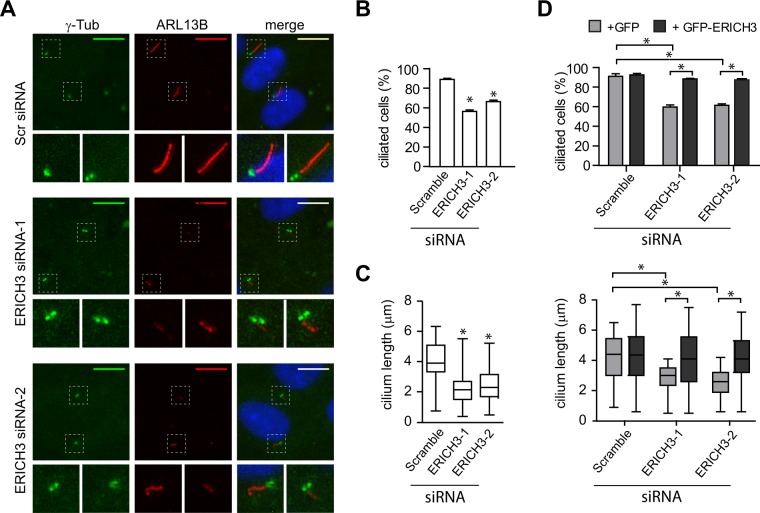
ERICH3 regulates ciliogenesis and ciliary length. (**A)** Representative images of hTERT-RPE1 cells treated with Scrambled (Scr) control or ERICH3 siRNAs. Cells were serum-starved for 24 h and stained for ciliary membrane (ARL13B; red) and centrosome (γ-tubulin; green) markers, and the nucleus (DAPI; blue). Small panels are higher magnification images of the boxed regions. Ciliary tip and basal body (bb) are indicated. Scale bars; 10 µm. **(B**–**D)** Quantification of cilium incidence and length in hTERT-RPE1 cells treated with Scrambled (Scr) control or ERICH3 siRNAs, serum starved for 24 h, and stained for cilia using ARL13B (B) or acetylated tubulin (**C,D**) antibodies. Panel D shows data for hTERT-RPE1 stably expressing GFP or GFP-ERICH3 (siRNA resistant) (related images to panel D shown in Supplementary Fig. S2C). Bar charts show mean ± SD (3 independent experiments; ~100 cells analysed per experimental condition). Box and whisker plots show data combined from 3 independent experiments (~50 cilia analysed per experimental condition); horizontal lines are 25, 50 and 75th percentiles; whiskers extend to maximum and minimum values. *p < 0.0001; Kruskal-Wallis test.

To investigate ERICH3 in non-motile human primary cilia, we employed the immortalised human retinal epithelial cell line (hTERT-RPE1), where cilium formation is induced by serum withdrawal. First we assessed if ERICH3 localises to primary cilia. Using a GFP-ERICH3 construct that is functional (described below in Fig. 2), we observed ERICH3 ciliary localisation in ~70% of transiently transfected cells (3 experiments; n = 100 cells per experiment), although the ciliary signals were faint (Supplementary Fig. S1A). To further examine GFP-ERICH3 ciliary localisation, we established stable hTERT-RPE1 cell lines expressing the construct. In these cells, GFP-ERICH3 shows an enriched localisation along the entire ciliary axoneme in 100% of analysed cells (3 experiments; n = 100 cells per experiment), as marked by acetylated alpha-tubulin (AcTub) staining (Fig. 1B). Co-staining for the centrosomal and basal body marker, gamma tubulin, revealed that some hTERT-RPE1 cells display a relatively weak GFP-ERICH3 signal at the ciliary base region (Supplementary Fig. S1B**)**. Together, our data from transient and stably expressing cells show that GFP-ERICH3 localises to the primary cilium.

### ERICH3 regulates cilium formation and length

To investigate if ERICH3 is required for cilium formation or structure, a ciliogenesis assay was performed on human hTERT-RPE1 cells treated with two independent siRNAs, each reducing ERICH3 expression by ~75% (Fig. S2A). An siRNA targeting CEP164 (CEP164-siRNA) was used as a positive control and a scrambled siRNA (Scr-siRNA) as a negative control. Following serum withdrawal, ~90% of cells treated with Scr-siRNA were ciliated (as measured by ARL13B staining), compared with only 26–30% ciliation for cells treated with the positive control (CEP164-siRNA) (Fig. 2A,B**;** Supplementary Fig. S2B). Compared to the negative control, cells treated with ERICH3-siRNA-1 or ERICH3-siRNA-2 showed a 35% and 26% reduction in ciliation, respectively (Fig. 2A,B). Next, by staining for the ciliary axonemal marker acetylated-alpha tubulin, we examined the ciliary length of ERICH3-depleted cells. Whereas Scr-siRNA-treated cells display a median cilium length of ~4.3 μm, the cilia of ERICH3-siRNA-treated cells are shorter, with median lengths of ~2.0 μm (Fig. 2A,C). To confirm the ERICH3 depletion phenotypes, we examined the effect of siRNA treatment on hTERT-RPE1 cells stably expressing the aforementioned GFP-ERICH3 construct, which was designed to be siRNA resistant (see methods). Cilia were stained with an acetylated tubulin antibody. Whereas ERICH3-siRNA treatment reduces ciliogenesis and cilium length in stable cell lines expressing GFP alone, these siRNA-induced phenotypes are almost fully rescued in stable cells expressing siRNA resistant GFP-ERICH3 (Fig. 2D**;** images in Supplementary Fig. S2C).

Together, these findings establish roles for ERICH3 in the regulation of cilium formation and length.

### ERICH3 depletion causes ciliary tip accumulation of IFT proteins

**Figure 3:**
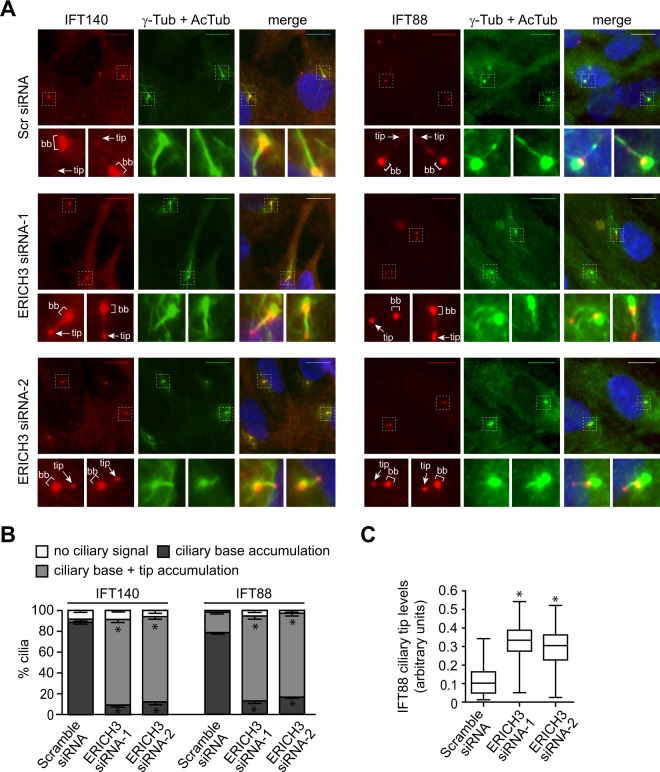
IFT proteins accumulate at the tips of ERICH3-depleted cilia. (**A)** Representative images and quantification of endogenous IFT88 (red) and IFT140 (red) ciliary localisations in hTERT-RPE1 cells treated with Scrambled (Scr) control or ERICH3 siRNAs. Ciliary axonemes stained with an acetylated tubulin (AcTub; green) antibody, centrosomes stained with a γ-tubulin (γ-Tub; green) antibody and nuclei stained with DAPI (blue). Small panels are higher magnification images of the boxed regions. Asterisk; ciliary base. Arrows; ciliary tip. Scale bars; 10 µm. **(B)** % cilia with the indicated IFT88 and IFT140 localisation phenotypes; data shows mean ± SD values (3 independent experiments; ~50 cilia analysed per experimental condition. *p < 0.05 (vs Scramble control); Kruskal-Wallis test. **(C**) Representative box and whisker plot (see Supplementary Fig. S3A for all 3 experiments; ~30 cilia analysed per experiment) of the ciliary tip levels of IFT88; horizontal lines are 25, 50 and 75th percentiles; whiskers extend to maximum and minimum values. *p < 0.0001 (vs Scramble control); Kruskal-Wallis test.

The ciliogenesis and cilium length defects associated with ERICH3 loss prompted us to examine possible roles for ERICH3 in IFT regulation. Specifically, we examined the endogenous ciliary localisations and distributions of IFT88 (IFT-B protein) and IFT140 (IFT-A protein) in ERICH3-depleted hTERT-RPE1 cells. For IFT88, control cells (Scr-siRNA) show prominent accumulations at the ciliary base, with weaker punctate signals along the ciliary axoneme, along with occasional low-level accumulations at the ciliary tip (Fig. 3A). In contrast, whilst IFT88 signals are retained at the base of ERICH3-depleted cells, prominent and elevated IFT88 accumulations are also observed at the ciliary tips of most cells (Fig. 3A–C**;** replicate datasets shown in Supplementary Fig. S3A). For IFT140 in control cells (Scr-siRNA), a prominent localisation is observed at the ciliary base, with little or mostly no detectable signal in the axoneme (Fig. 3A). In ERICH3-siRNA-treated cells a prominent accumulation of IFT140 occurs at the ciliary tip, as well as the ciliary base (Fig. 3A,B). Thus, both IFT88 and IFT140 abnormally accumulate at the ciliary tips of ERICH3-depleted cells, which is suggestive of a defect in IFT machinery turnaround at the ciliary tip.

### ERICH3 controls the ciliary levels of retrograde IFT regulators ARL13B and BBS5

**Figure 4:**
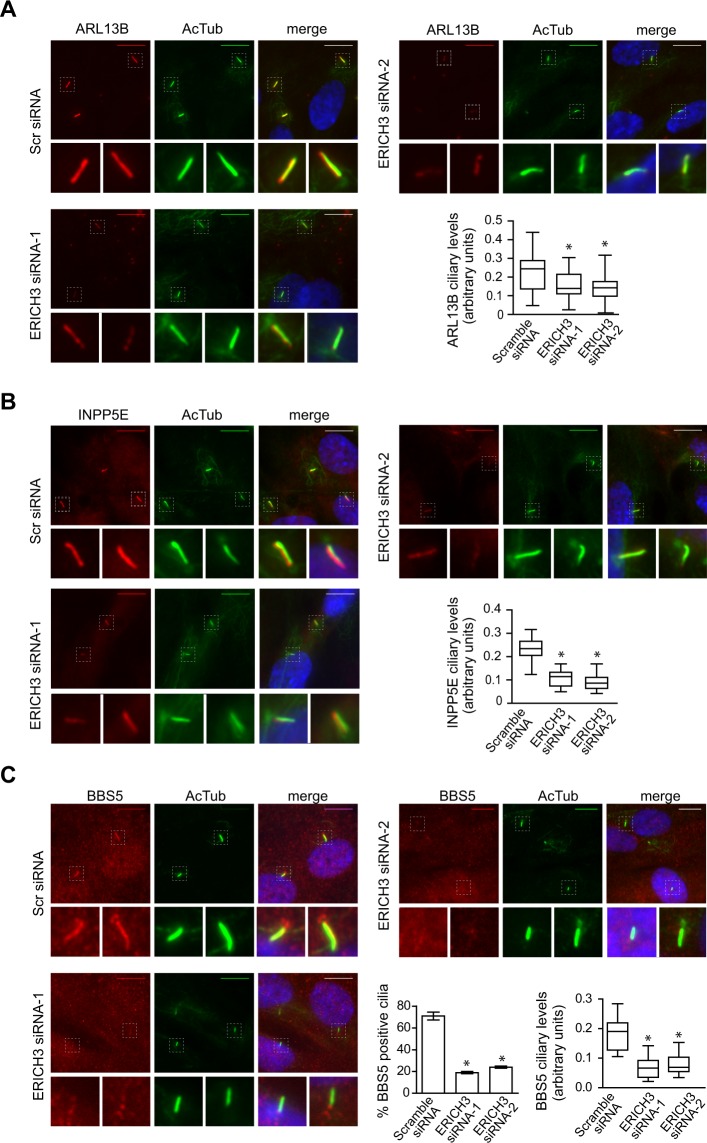
ARL13B, INPP5E and BBS5 levels are reduced in ERICH3-depleted cilia. Representative images and quantification of endogenous ARL13B, INPP5E and BBS5 ciliary levels in hTERT-RPE1 cells, treated with Scrambled (Scr) control or ERICH3 siRNAs. Ciliary axonemes stained with an acetylated tubulin (AcTub; green) antibody and nuclei stained with DAPI (blue). Small panels are higher magnification images of the boxed regions. Scale bars; 10 µm. Data in box and whisker plots are from representative experiments (see Supplementary Fig. S3B,C for all 3 experiments; ~30 cilia analysed per experiment); horizontal lines are 25, 50 and 75th percentiles; whiskers extend to maximum and minimum values. Note that the individual data points in the box and whisker plots are mean ciliary intensity values. Data in bar chart are means ± SD (3 independent experiments; ~100 cells analysed per experimental condition). *p < 0.005 (vs Scramble control); Kruskal-Wallis test.

To further examine a potential retrograde IFT-associated role for ERICH3, we assessed if ERICH3 is required for the ciliary localisations of proteins involved in retrograde IFT regulation. First, we examined the ciliary membrane-associated G-protein ARL13B, which regulates the retrograde trafficking of IFT machinery from the ciliary tip of hTERT-RPE1 cells^46^. We found that compared to controls (Scr-siRNA), the mean signal intensity of endogenous ARL13B at the ciliary membrane is significantly reduced in ERICH3-depleted cells (Fig. 4A; replicate datasets shown in Supplementary Fig. S3B). Since ARL13B regulates the ciliary targeting of INPP5E in RPE1 cells^46,49^, we also investigated INPP5E localisations. Similar to our ARL13B observations, the mean ciliary signal intensity of endogenous INPP5E is reduced in cells lacking ERICH3 (Fig. 4B; replicate datasets shown in Supplementary Fig. S3C). Therefore, ERICH3 regulates the ciliary localisation of ARL13B and a proposed effector of this G-protein, INPP5E.

Next, we wondered if ERICH3 regulates the ciliary levels of BBSome components, which facilitate the removal of multiple proteins from cilia and flagella via its association with retrograde IFT^33–35,37,39,40,63,75^. Specifically, we examined BBS5, which associates with a stable core of the BBSome^38^. In control cells (Scr-siRNA), ~75% of cilia are positive for BBS5, which is found along the entire cilium length and as punctate signals at the basal body region (Fig. 4C). In contrast, only ~20% of ERICH3-depleted cells display detectable BBS5 signals in cilia; also, when detectable in the cilium, the ciliary BBS5 levels are reduced compared to control cells. (Fig. 4C). The defective BBS5 localisation phenotypes are rescued in cells expressing the siRNA resistant GFP-ERICH3 construct, but not GFP alone (Supplementary Fig. S4).

Thus, ERICH3 positively regulates the ciliary levels of multiple proteins associated with retrograde IFT.

### ERICH3, ARL13B and the BBSome display interdependency for their targeting to cilia

**Figure 5:**
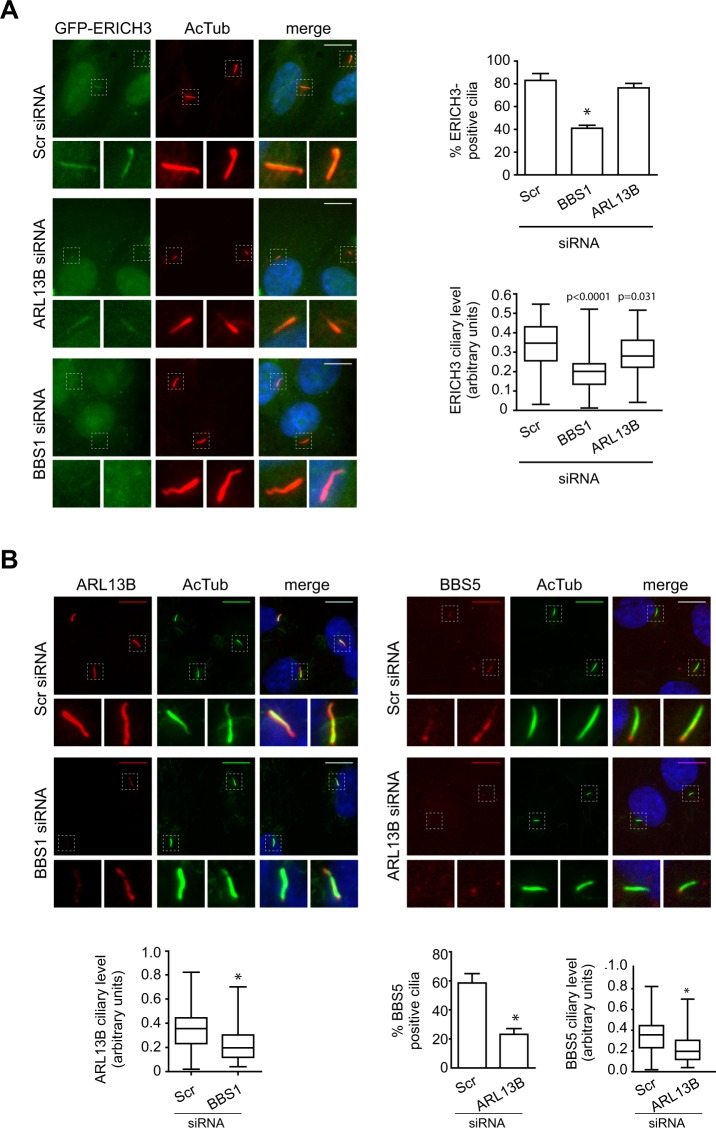
ERICH3 ciliary levels are regulated by interdependent ARL13B and BBS1. Representative images and quantification of ciliary GFP-ERICH3 (**A**), ARL13B or BBS5 (**B**) in hTERT-RPE1 cells treated with Scrambled (Scr) control, BBS1 or ARL13B siRNAs. Ciliary axonemes stained using an acetylated tubulin antibody (AcTub; red in (**A**) and green in (**B**)) and nuclei stained with DAPI (blue). Small panels are higher magnification images of boxed regions. Scale bars; 10 µm. Box and whisker plot data is from one representative experiment (see Supplementary Fig. S3D–F for all 3 experiments; ~30 cilia analysed per experiment); horizontal lines are 25, 50 and 75th percentiles; whiskers extend to maximum and minimum values. Data in bar charts show mean ± SD (3 independent experiments; ~100 cells analysed per experimental condition). *p < 0.0001 (vs Scramble siRNA); Kruskal-Wallis test.

The requirement of ERICH3 for ensuring normal ciliary localisation of ARL13B and BBS5 prompted us to investigate if ARL13B and the BBSome are reciprocally required for targeting ERICH3 to cilia. For these experiments, we employed hTERT-RPE1 cells stably expressing GFP-ERICH3 and siRNAs against ARL13B and the BBSome subunit, BBS1. The siRNAs deplete ARL13B and BBS1 RNA levels by >80% (Supplementary Fig. S5). In BBS1-depleted cells, we observed strong disruption of ciliary ERICH3. Specifically, the frequency of GFP-ERICH3-positive cilia was reduced by ~50% in BBS1-disrupted cells compared with control cells (Scr-siRNA); in addition, for those cilia that were GFP-ERICH3-positive, the signal intensity was reduced by ~40% in cells depleted of BBS1 (Fig. 5A; replicate datasets in Supplementary Fig. S5C). Whilst ARL13B depletion did not affect the frequency of GFP-ERICH3-positive cilia, we did observe a modest reduction (~18%) in ERICH3 ciliary levels (Fig. 5A; replicate datasets in Supplementary Fig. S3D**)**. Thus, BBS1, and to a lesser extent ARL13B, are required for normal ERICH3 ciliary levels.

Since BBS1 and ARL13B are reciprocally required for targeting ERICH3 to cilia, we wondered if the BBSome and ARL13B are themselves interdependent for their ciliary localisations. We found that BBS1 depletion causes a very significant reduction (~45%) in endogenous ARL13B ciliary levels (Fig. 5B; replicate datasets in Supplementary Fig. S3E**)**. In the reverse experiment we assessed the localisation of the BBSome using a BBS5 antibody and observed a large reduction in the percentage of detectable BBS5-positive cilia in ARL13B-depleted cells; also, in those cilia positive for BBS5, the levels of BBS5 are reduced (Fig. 5B**;** replicate datasets for BBS5 cilium levels are shown in Supplementary Fig. S3F). Therefore, the ciliary localisations of ARL13B and the BBSome are interdependent.

Together with the data presented above in Fig. 4, our findings show that ERICH3, ARL13B and the BBSome are interdependent in regulating their respective ciliary levels, although the requirement of ARL13B for ERICH3 localisation is somewhat modest.

### The removal of Sonic hedgehog signaling proteins from cilia depends on ERICH3

**Figure 6:**
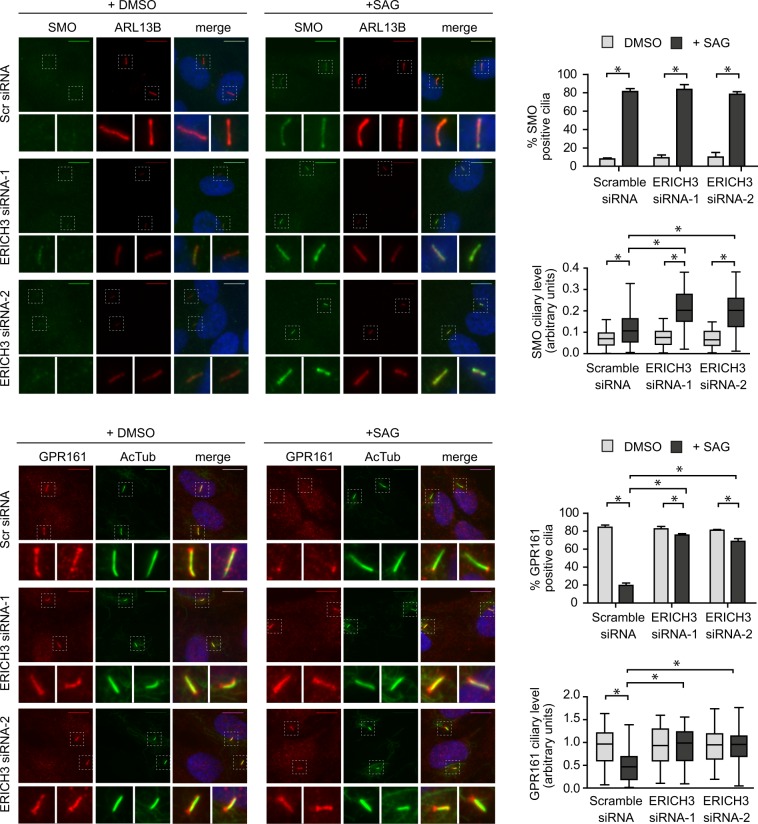
ERICH3 regulates the ciliary levels of Smoothened and GPR161. Representative images and quantification of endogenous ciliary Smoothened (SMO; green) and GPR161 (red) levels in hTERT-RPE1 cells treated with Scrambled (Scr) or ERICH3 siRNAs, under basal conditions (+DMSO) and conditions that stimulate the Shh pathway (+SAG). Cilia stained either with acetylated tubulin (AcTub; green) or ARL13B (red) antibodies, and nuclei stained with DAPI (blue). Small panels are higher magnification images of the boxed regions. Scale bars; 10 µm. Data in bar charts are mean ± SD (3 independent experiments; ~100 cells analysed per experimental condition). Data in box and whisker plots are from a representative experiment (see Supplementary Fig. S3G,H for all 3 experiments; ~100 cilia analysed per experimental condition); horizontal lines are 25, 50 and 75th percentiles; whiskers extend to maximum and minimum values. *p < 0.0001; Kruskal-Wallis test.

Retrograde IFT controls cilium-based Sonic hedgehog (Shh) signaling by preventing ciliary accumulation of positive signal regulators (eg. Smoothened) when the pathway is off and promoting ciliary removal of negative signal regulators (eg. GPR161) when the pathway is activated^13,35,37,40,46,63^. Given the evidence above that ERICH3 serves a role in retrograde IFT-associated pathways, we wondered if ERICH3 regulates SMO and GPR161 ciliary levels. To stimulate Shh signaling we employed the Smoothened agonist SAG, which has been used by multiple studies to turn on the pathway in RPE1 cells^13,40,46^. In control cells (Scr-siRNA), most cilia are SMO-negative and GPR161-positive under basal conditions (DMSO vehicle control); upon SAG treatment, these phenotypes reverse due to the ciliary accumulation of SMO and ciliary removal of GPR161 (Fig. 6**;** replicate datasets shown in Supplementary Fig. S3G,H). In ERICH3-depleted cells, the ciliary levels of SMO and GPR161 under basal conditions are unaffected (Fig. 6**;** replicate datasets shown in Supplementary Fig. S3G,H). However, treatment of these cells with SAG results in abnormally high ciliary levels of SMO; in addition, most cilia abnormally retain GPR161 signals at levels comparable to those observed under basal conditions (Fig. 6**;** replicate datasets shown in Supplementary Fig. S3G,H**)**. Thus, ERICH3 is required for removing GPR161 from cilia and limits the ciliary levels of SMO under conditions of Shh pathway activation.

## Discussion

IFT and associated cargo adaptors are thought to be critical for regulating the ciliary levels of various signaling molecules that function within the organelle. This is best understood for the Shh pathway where various IFT-A, IFT-B and BBSome-associated proteins are linked to the ciliary targeting and removal of positive (eg. SMO) and negative (eg. PTCH1, GPR161) pathway regulators. In the present study, we identify ERICH3 as a component of the primary cilium and describe roles for ERICH3 in regulating the ciliary levels of Shh signaling proteins. Specifically, in Shh pathway-stimulated cells depleted of ERICH3, SMO and GPR161 ciliary levels are abnormally high. Consistent with a role for ERICH3 in removing signaling proteins from cilia, ERICH3-disrupted cells display ciliary tip accumulation of IFT proteins, along with reduced ciliary levels of retrograde IFT regulators ARL13B, INPP5E and the BBSome. We also describe an interdependent relationship between ERICH3, ARL13B and the BBSome in the regulation of their ciliary levels. Thus, our work places the mostly uncharacterised ERICH3 protein in the primary cilium, where it interacts with pathways involved in retrograde IFT-cargo regulation.

We observed a high level of GFP-ERICH3 signal in the primary cilia of hTERT-RPE1 cells stably expressing this construct. This finding is in agreement with a recent paper that used proteomics and immunohistochemistry to show that ERICH3 is an abundant protein in motile cilia of human airway epithelial (HAE) cells^74^. Furthermore, the HAE cell study showed that the ERICH3 protein is not detectable in undifferentiated non-ciliated cells^74^. Together with a previous finding from our group that ERICH3 is co-expressed with ciliary genes^71^, we conclude that ERICH3 is strongly associated with both motile *and* primary cilia. Interestingly, ERICH3 ciliary distributions differ between these ciliary subtypes; whereas ERICH3 localises mostly to the proximal half of HAE motile cilia^74^, ERICH3 decorates the entire length of hTERT-RPE1 primary cilia. Whilst the reasons for differential ciliary localisation of ERICH3 is unknown, one possibility relates to underlying differences between motile vs non-motile ciliary structures. Interestingly, ARL13B, which we identify in this study as a functional interactor of ERICH3 (discussed below), also displays full length vs proximal ciliary distributions in motile and non-motile cilia, respectively^45,47^. Whether ERICH3 associates with the ciliary membrane, microtubules or cytosol remains to be determined.

Our data implicates a role for ERICH3 in regulating the ciliary levels of Shh signaling proteins. In wild type cells, Shh pathway stimulation increases the ciliary levels of SMO (positive pathway regulator) and decreases the ciliary levels of GPR161 (negative pathway regulator), ostensibly on account of enhanced trafficking of these proteins into and out of the organelle. However, in ERICH3-depleted cells treated with the SMO agonist, SMO ciliary levels are abnormally high and GPR161 fails to be removed from the organelle. These findings are strikingly similar to what has been reported for cells with disrupted retrograde IFT-cargo regulation. For example, in cells lacking the IFT-A non-core subunit IFT139, abnormally high numbers of SMO- and GPR161- positive cilia occur in Shh pathway stimulated and unstimulated conditions^13^. Similarly, SMO ectopically localises to cilia in unstimulated BBS1, BBS3 and BBS7 knockout cells; ciliary levels increase even further when the Shh pathway is stimulated and a reduced pathway activation is observed at least for BBS7-disrupted cells^37,40,63,66^. Ectopic ciliary SMO is also reported in cells lacking negative regulators of ciliary BBSome levels, namely the IFT25/27 dimer and the BBSome-binding protein LZTFL1; additionally, IFT27 loss reduces Shh pathway activation^62,63,76,77^. Furthermore, elevated GPR161 ciliary levels occur in stimulated and unstimulated cells lacking BBS3^37^. Finally, in ARL13B disrupted cells, where IFT-A/B proteins accumulate in the cilium, Shh pathway activation causes elevated SMO in cilia and a failure to remove GPR161 from the cilium; ARL13B-disrupted fibroblasts also show ectopic SMO in unstimulated cells and exhibit reduced Shh pathway activity^46,50,78^. Thus, the ERICH3 depletion phenotype for ciliary SMO/GPR161 phenocopy those of retrograde IFT disrupted cells. There is one important distinction, however, which is that SMO remains excluded from ERICH3-depleted cilia in unstimulated conditions, whereas this is not the case for most cells lacking the aforementioned genes. Therefore, ERICH3 controls ciliary SMO and GPR161 levels in RPE1 cells only in the context of Shh pathway activation. Notably, ARL13B appears to function in a similar manner in RPE1 cells^46^.

Our finding of mislocalised IFT-A/B, BBSome and ARL13B proteins in ERICH3-depleted cells supports the notion that ERICH3 functions within retrograde IFT pathways to facilitate the ciliary removal of SMO and GPR161. Specifically, ERICH3 loss causes a characteristic retrograde IFT defect, namely the accumulation of IFT-A and IFT-B proteins at the ciliary tip. In addition, ERICH3-depleted cells display reduced ciliary levels of a BBSome retrograde IFT cargo adaptor component (BBS5) and lower ciliary intensity of ARL13B, which regulates IFT turnaround at the ciliary tip. A role for ERICH3 in retrograde IFT regulation is also supported by the cilium length and modest ciliogenesis defects we observe in ERICH3-depleted cells, and by the ciliary localisation interdependencies we identified for ERICH3, ARL13B and the BBSome. Indeed, the hTERT-RPE1 ciliogenesis phenotype may relate directly to ERICH3’s role in regulating the localisation of IFT140, given reports that IFT-A core complex proteins (IFT122/140) are reported to control cilium incidence and number in this cell type^19,79^.

One mechanistic possibility to explain the various phenotypes we observed is that ERICH3’s primary role is to regulate the ciliary targeting of the BBSome. However, this function is countered by recent findings of normal IFT-A/B protein localisations in BBS1 knockout RPE1 cells (versus disrupted IFT-A/B protein localisations in ERICH3-disrupted cells), although findings in *C. elegans* implicate BBS genes in retrograde IFT train organisation at the ciliary tip^40,80^. Alternatively, ERICH3 may regulate the IFT-A retrograde machinery directly or indirectly via the reported role of ARL13B in preventing IFT protein accumulation at the ciliary tip^46^. Indeed, as mentioned above, both ERICH3 and ARL13B localise to proximal regions of motile cilia^47,74^, and ARL13B (like ERICH3) controls ciliary SMO and GPR161 levels only during Shh pathway activation, at least in RPE1 cells^46^. A role for ERICH3 in directing ciliary tip turnaround of the retrograde IFT machinery does, however, raise the question as to why BBSome protein levels are reduced in ERICH3-depleted cells, rather than increased as the model might predict. Thus, although our data strongly links ERICH3 function to retrograde IFT associated pathways, the precise mechanism involved remains to be determined. Future work to identify the biochemical interactors of ERICH3 should shed light on its mechanism of action and the nature by which the protein interacts with retrograde IFT machinery and/or its regulators. It is notable that ERICH3’s even (non-punctate) distribution along the length of the hTERT-RPE1 cilium is not consistent with that expected of a protein undergoing IFT; thus, ERICH3’s role in regulating retrograde IFT may not involve the protein’s association with moving IFT trains.

In conclusion, we have identified ERICH3 as a new component of the primary cilium and shown that it interacts with retrograde IFT-associated pathways to control the ciliary levels of Shh signaling molecules. Given that the functional interactors of ERICH3 are all associated with ciliary diseases such as Joubert syndrome (ARL13B, INPP5E) and Bardet Biedl syndrome, ERICH3 represents an attractive candidate for mutations in unresolved ciliopathies. Furthermore, our finding of a ciliary role for ERICH3 raises the possibility that ciliary dysfunction may at least partially underlie reported associations of ERICH3 with major depression disorder, osteoporosis and colorectal cancer^72,73^.

## Materials and Methods

### Cell culture and transfection

hTERT-RPE1 cells were grown at 37 °C under 5% CO_2_ in DMEM/F12 media (Sigma Aldrich) containing 10% fetal bovine serum (FBS; Biosciences), 2 mM L-Glutamine, and 0.348% sodium bicarbonate. Cilium formation was induced by serum starvation for 48 h. For transient transfection, cells were grown to 90% confluency and transfected with plasmid DNA using using TransIT-X2® as per the manufacturer’s instructions. Stable hTERT-RPE1 cell lines were generated by transfecting plasmids using TransIT-X2® transfection Reagent and selecting clones with 500 µg/ml G418 (Sigma Aldrich). For depletion experiments, hTERT-RPE1 cells were transfected with Silencer Select siRNAs (Ambion) using Lipofectamine 2000 (Thermo-Fisher). After 16 h, cells of 90% confluence were serum-starved for 48 h to induce ciliogenesis. siRNAs are listed in Supplementary Table S1.

### Quantitative PCR (qPCR)

*ERICH3*, *ARL13B*, and *BBS1* mRNA levels following siRNA-mediated depletion were measured by qPCR. Total RNA was purified with an InviTrap®Spin Cell RNA Mini kit (Stratec Molecular) and concentration measured using a Nano-Drop 2000 spectrophotometer. cDNA was synthesised with a High Capacity cDNA Reverse Transcription Kit (Applied Biosystems) using 200–500 ng of RNA per reaction. qPCR reactions were performed using SYBR green detection in a LightCycler 480 II Real-Time PCR instrument (Roche Life Sciences). The annealing temperature was 60 °C for all triplicate reactions. *ERICH3*, *BBS1*, and *ARL13B* mRNA levels were normalised against GAPDH mRNA levels, employing the ΔΔCt method^81^. Primers are listed in Supplementary Table S1.

### Immunofluorescence staining

hTERT-RPE1 cells grown on glass coverslips were washed three times with phosphate-buffered saline (PBS). For ARL13B, acetylated tubulin, γ-tubulin, SMO and GPR161 antibody staining, cells were fixed with 3% paraformaldehyde (PFA) at 37 °C for 10 min., and permeabilised in 0.2% Triton X-100/PBS (1% for SMO and GPR161 staining) at 37 °C for 5 min. For IFT88 and IFT140 antibody staining, cells were fixed and permeabilised by incubating in methanol at −20 °C for 5 min, followed by dipping into cold acetone and subsequent 10 min. drying on filter paper. All samples were blocked for 30 min at 37 °C with 3% BSA (bovine serum albumin) in 0.2% Triton X-100/PBS. Blocking and subsequent steps were performed in a moist chamber to avoid sample drying. Cells were incubated with primary antibodies (diluted in PBX blocking solution; see Supplementary Table S1 for the specific dilution factors employed) for 1–2 h at room temperature. After 3 × 5 min washes with PBS, cells were incubated in the dark for 1–2 h at room temperature with secondary antibodies conjugated to fluorescent dyes (diluted in PBX). All antibodies used in this study are listed in Supplementary Table S1. For DNA staining, DAPI (4′,6-Diamidino-2-phenylindole, Sigma-Aldrich) was included with the secondary antibodies. Samples were washed three times with PBS, and coverslips mounted on glass slides in Mowiol (Sigma-Aldrich).

### Microscopy and image analysis

Widefield images were taken as z-stacks at 0.4 µm intervals using a Zeiss AxioImager M1 microscope equipped with a Zeiss Plan Neofluar 40x/NA1.3 oil immersion objective and a QImaging Retiga R6 CCD camera. Cilia length and fluorescence intensity measurements were performed using Fiji/ImageJ (NIH)^82^ on widefield images from cells stained with a cilia marker (acetylated tubulin or ARL13B). For ciliary length measurements, the region of interest was manually defined using the line segment tool. For ciliary intensity measurements (ARL13B, INPP5E, BBS5, SMO and GPR161), acetylated tubulin or ARL13B signals were first used to segment the ciliary axoneme and background subtraction was performed using the same sized region of interest as the segmented cilium. Average pixel intensity was determined for the protein of interest using the Measure Object Intensity module. For the ciliogenesis assay, cells stained for the ciliary marker ARL13B were randomly selected and analysed from multiple field areas of 2–4 coverslips per condition. All statistical analyses were performed using Prism software.

### Generation of GFP-ERICH3 recombinant plasmids

ERICH3 cDNA was purchased from Lifesciences as GeneCube constructs. cDNA was amplified from the vectors using primers incorporating cloning restriction sites to XhoI and BamHI. PCR products and GFP vector (pEGFP-N1) were digested with restriction enzymes and then ligated for 1 hr at RT with Quick-Stick Ligase at 1:3 molar (vector:insert) ratios. Ligations containing pEGFP-N1-ERICH3 were transformed into chemically competent DH5alpha *E. coli* and transformed cells selected via kanamycin. To identify colonies containing plasmids with the insert of interest, individual colonies were placed into a PCR tube containing the prepared Touchdown PCR reagents, followed immediately by the PCR reaction. Point mutations for the siRNA-resistant pEGFP-N1-ERICH3 construct were inserted by site-directed mutagenesis (QuikChange kit; Agilent Technologies) using primers indicated in Supplementary Table S1.

## Supplementary information

Supplemental Information
