# Supplementary material for: ERICH3 in Primary Cilia Regulates Cilium Formation and the Localisations of Ciliary Transport and Sonic Hedgehog Signaling Proteins
Source: Sci Rep. 2019 Nov 11;9:16519. doi: 10.1038/s41598-019-52830-1 (PMC6848114; doi:10.1038/s41598-019-52830-1)
Supplement: Supplementary file 1 — Supplemental Information [file 41598_2019_52830_MOESM1_ESM.pdf]

Supplementary information for:

ERICH3 IN PRIMARY CILIA REGULATES CILIUM FORMATION AND THE LOCALISATIONS OF CILIARY TRANSPORT AND SONIC HEDGEHOG SIGNALING PROTEINS

Mona Alsolami, Stefanie Kuhns, Manal Alsulami, & Oliver E. Blacque

School of Biomolecular and Biomedical Science, University College Dublin, Belfield, Dublin 4, Ireland

Supplementary Table S1
Supplementary Figure S1
Supplementary Figure S2
Supplementary Figure S3
Supplementary Figure S4
Supplementary Figure S5

Supplementary Table S1

Antibodies used in this study

Antibodies	Source	Catalog numbers	Dilution (IF)
Monoclonal mouse anti-acetyl-α-tubuli	Gift from G. Pereira	C3B9	1:250
Monoclonal mouse anti-γ-tubulin	Sigma-Aldrich	GTU88	1:1000
Polyclonal rabbit anti-ARL13B	Proteintech	17711-1-AP	1:3000
Polyclonal rabbit anti-INPP5E	Proteintech	17797-1-AP	1:500
Polyclonal rabbit anti-TULP3	Proteintech	13637-1-AP	1:100
Polyclonal rabbit anti-IFT140	Proteintech	17460-1-AP	1:100
Polyclonal rabbit anti-IFT88	Proteintech	13967-1-AP	1:500
Polyclonal rabbit anti-GPR161	Proteintech	13398-1-AP	1:500
Monoclonal mouse anti-SMO	Santa Cruz	sc-166685	1:100
Polyclonal rabbit anti-BBS5	Proteintech	14569-1-AP	1:500
Secondary-DAPI	Thermofisher	D1306	1:5000
Alexa Fluor Plus 488	Thermofisher	A32723, A27034SAMPLE	1:500
Alexa Fluor 568	Thermofisher	A-11004, A-11011	1:500
Alexa Fluor Plus 647	Thermofisher	A32733	1:500

Oligo DNAs used in this study

Names	Sequences
ERICH3-FW	5'-AAAACTCGAGCTATGCTTGCCAAGGGCAAGAA-3'
ERICH3-Rev	5'-TTTGGATCCCGTCAACGGCCATACTTACCAC-3'
qPCR ERICH3-F	5'-ATGGGTGCTTTCCATGCTT-3'
qPCR ERICH3-R	5'-TTCTCTGTGAATTGCCTATGGA-3'
qPCR GAPDH-F	5'-AGCCACATCGCTCAGACAC-3'
qPCR GAPDH-R	5'-GCCCAATACGACCAAATCC-3'
qPCR ARL13B-F	5'-CTGTGCTCCTGAGAGTCCAA-3'
qPCR ARL13B-R	5'-GAAGTCTAGTGACTTTAGGGGTTCC-3'
qPCR BBS1-F	5'-GCCATCCTGACCATGAACCTC-3'
qPCR BBS1-R	5'-AAAGCAAAGGCTGGTCACTGC-3'

Plasmid vectors used in this study

Vectors	Insert
pEGFP-N1	Human ERICH3

siRNAs used in this study

Name	siRNA ID (Ambion) or sequence
Scramble	s813
CEP164	GGUGACAUUUACUAUUUCATT
ERICH3-1	s43148
ERICH3-2	s43149
ARL13B	s47283
BBS1	s1892

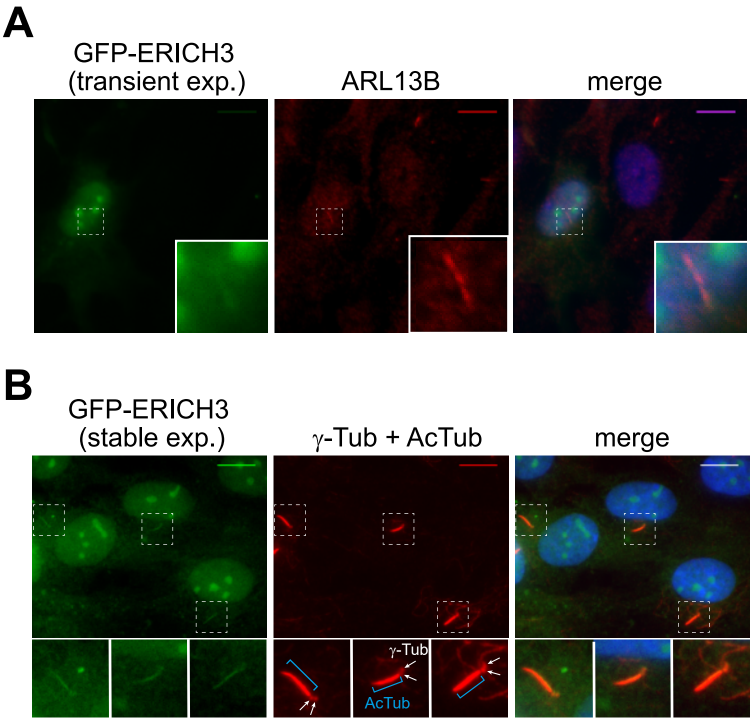

Supplementary Figure S1 (linked to Figure 1). Localisation of transiently transfected and stably-expressed GFP-ERICH3 in hTERT-RPE1 cells. **(A)** Representative images of hTERT-RPE1 cells transiently transfected with GFP-ERICH3. Cells were serum starved for 48 h, and stained for cilia (ARL13B; red) and the nucleus (DAPI; blue). Scale bars; 10 µm. Insets are higher magnification images of the boxed regions. **(B)** Representative images of hTERT-RPE1 cells stably expressing GFP-ERICH3. Cells were serum starved for 48 h, and stained for cilia (acetylated tubulin; AcTub; red), the centrosome (γ-tubulin; γ-Tub; red) and the nucleus (DAPI; blue). Scale bars; 10 µm. Insets are higher magnification images of the boxed regions. Arrows denote the mother and daughter pericentriolar signals for γ-Tub at the ciliary base.

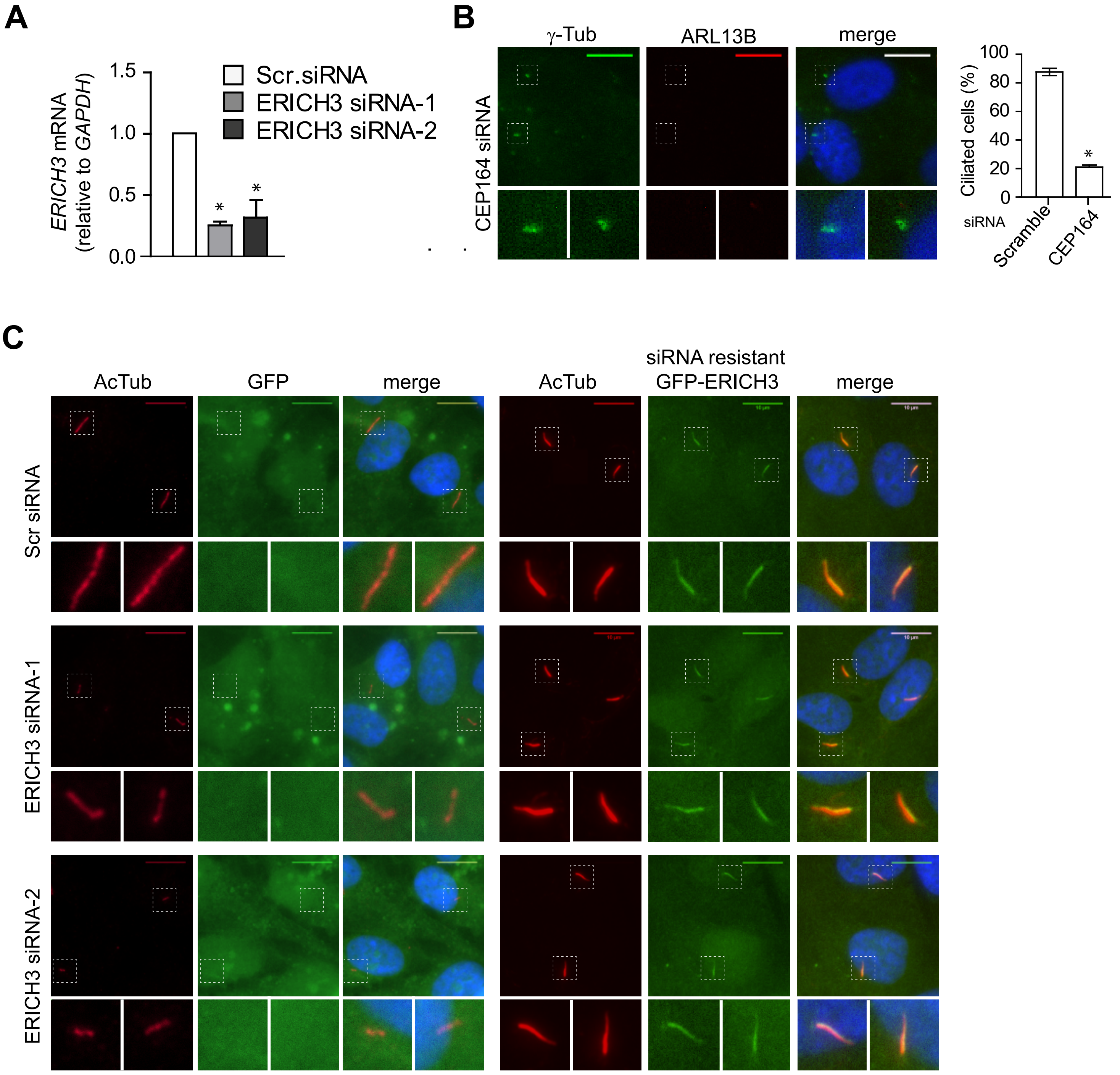

Supplementary Figure S2 (linked to Figure 2). Efficiency of siRNA-mediated depletion of ERICH3, and cilium formation and structure phenotypes in depleted cells. **(A)** qPCR data from hTERT-RPE1 cells transfected with two independent siRNAs targeting human ERICH3 (siRNA-1, siRNA-2). Graphs show relative mRNA levels remaining compared to cells transfected with non-targeting Scrambled (Scr) siRNA control (Neg). Data are means ± S.D. of 3 independent experiments. *p<0.001; unpaired t-test. **(B)** Representative images of hTERT-RPE1 cells treated with CEP164 siRNA (positive control for experiment in Fig. 2B). Cells were serum-starved for 48 h and stained for ciliary membrane (ARL13B; red) and centrosome (γ-tubulin; green) markers, and the nucleus (DAPI). Small panels are higher magnification images of the boxed regions. Scale bars; 10 µm. Bar charts show mean ± SD (3 independent experiments; 100 cells analysed per experimental condition). *p<0.001; unpaired t-test (2-tailed). **(C)** Representative images of hTERT-RPE1 cells stably expressing either GFP alone or siRNA resistant GFP-ERICH3. Cells were treated with Scrambled (Scr) control or ERICH3 siRNAs, serum starved for 24 h, and stained for cilia using an acetylated tubulin (AcTub; red) antibody and the nucleus using DAPI (blue). Small panels are higher magnification images of the boxed regions. Scale bars; 10 µm.

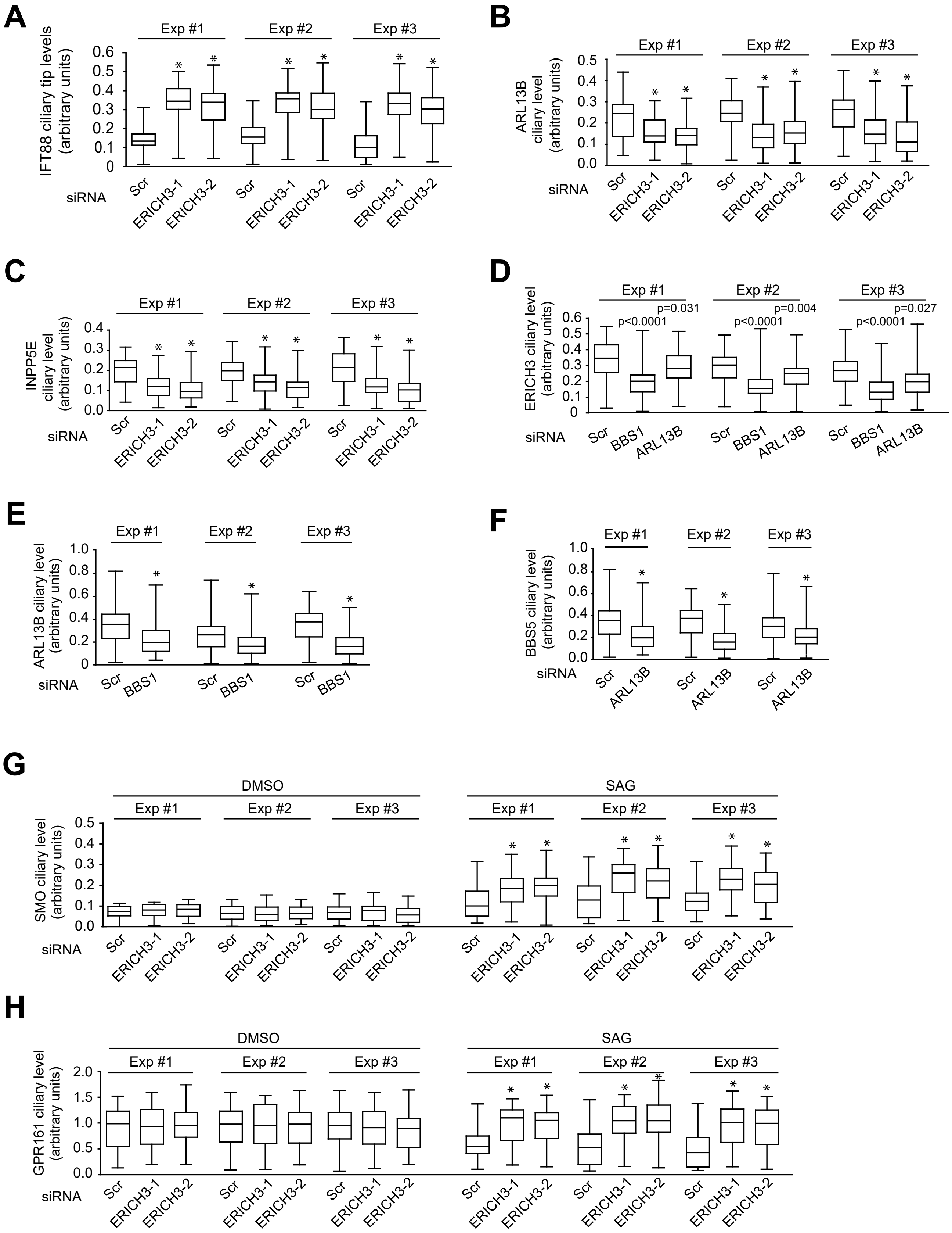

Supplementary Figure S3 (linked to Figures 4-6). **(A-G)** Data from the 3 independent experiments linked to the representative box and whisker plots shown in Figures 4, 5 and 6. * $p<0.0001$ (vs DMSO controls); Kruskal-Wallis test.

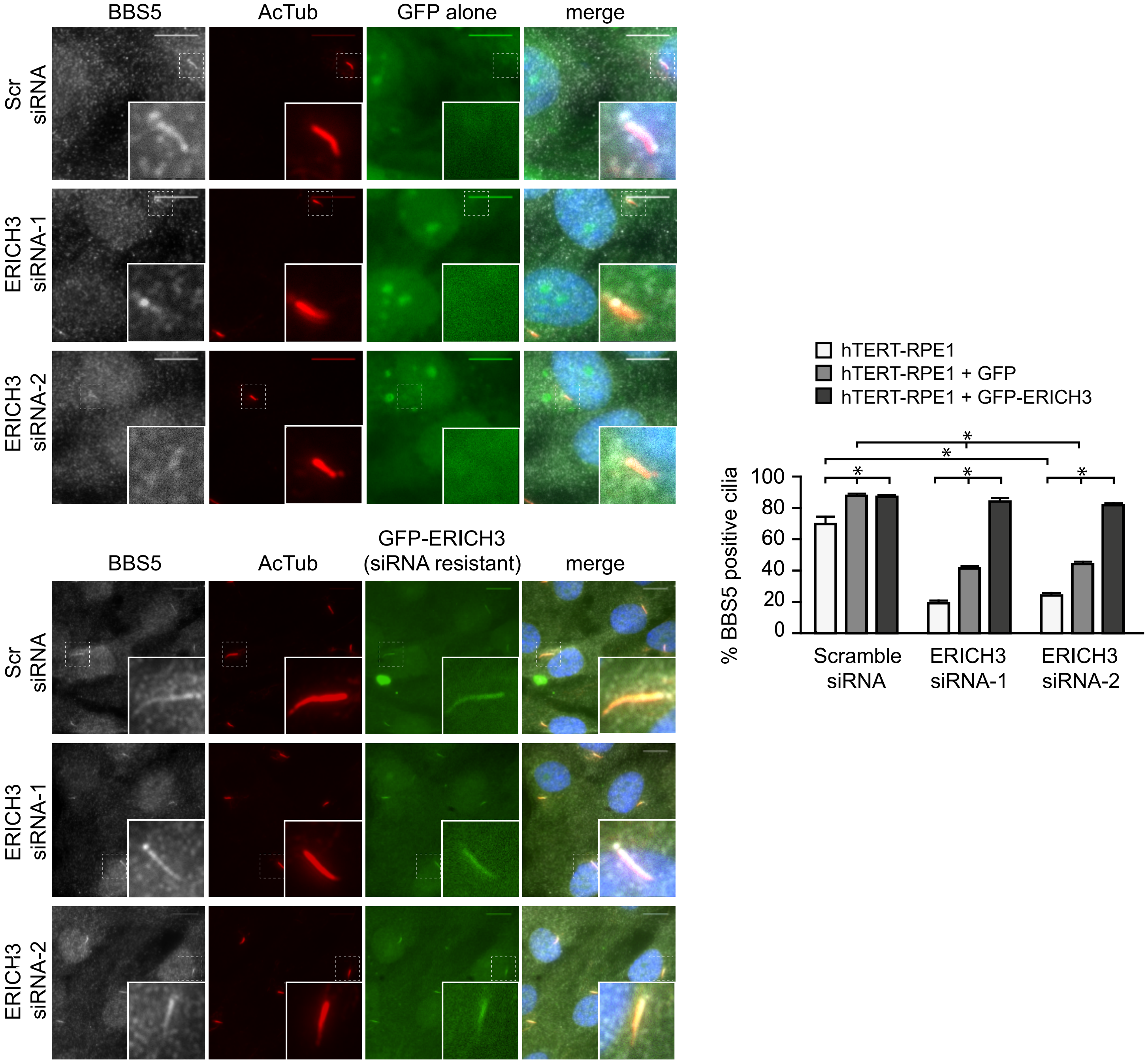

Supplementary Figure S4 (linked to Figure 4). Ciliary localisations of BBS5 in hTERT-RPE1 cells. Representative images and quantifications of endogenous BBS5 ciliary localisation in hTERT-RPE1 cells stably expressing either GFP alone or siRNA resistant GFP-ERICH3. Cells were treated with Scrambled (Scr) control or ERICH3 siRNAs, serum starved for 24hr, and stained with antibodies against acetylated tubulin AcTub; red), BBS5 (gray); nucleus stained using DAPI (blue). Insets are higher magnification images of the boxed regions. Scale bars; 10 µm. Bar chart show means ± SD (3 independent experiments; ~100 cells analysed per experimental condition). *p<0.001; unpaired t-test (2-tailed).

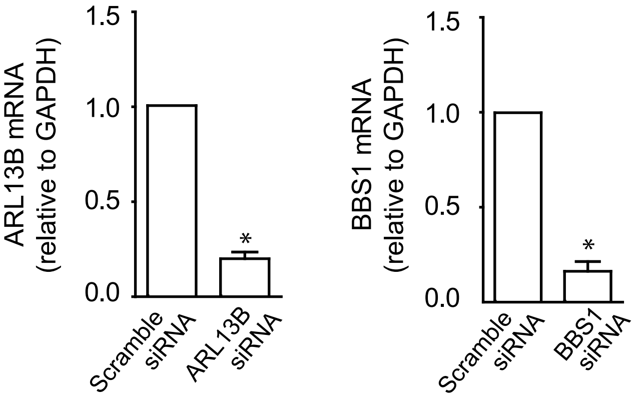

*

Supplementary Figure S5 (linked to Figure 5). qPCR data from hTERT-RPE1 cells transfected with siRNAs targeting human ARL13B and BBS1. Graphs show relative mRNA levels remaining compared to cells transfected with non-targeting Scrambled (Scr) siRNA control. Data are means ± S.D. of 3 independent experiments. ARL13B and BBS1 siRNA data normalised to the Scramble siRNA. *p<0.001 (vs Scramble siRNA); unpaired t-test (2-tailed).
